# mRNA-based immunotherapy improves the efficacy of adoptively transferred TCR transgenic T cells in a murine solid tumour model

**DOI:** 10.1038/s41598-026-65120-4

**Published:** 2026-08-08

**Authors:** Janna Krueger, Janne J. Ruotsalainen, Johannes Lutz, Regina Heidenreich

**Affiliations:** CureVac SE, Friedrich-Miescher-Straße 15, 72076 Tübingen, Germany

**Keywords:** mRNA, Immunotherapy, Adoptive T cell therapy—ACT, Transgenic T cell receptor (TCR), Cancer immunotherapy, Cancer vaccine, Cancer, Immunology, Oncology

## Abstract

Adoptive T cell therapy (ACT) has shown remarkable clinical success in treating haematological malignancies; however, its efficacy against solid tumours remains limited. This is largely due to poor persistence and functionality of transferred T cells, restricted tumour infiltration, and the presence of an immunosuppressive tumour microenvironment. Here, using the MC38-OVA murine tumour model, we tested whether combining Transgenic T cell receptor (TCR^tg^) T cell therapy with an adjunctive mRNA-based immunotherapy could address these challenges and drive effective and long-lasting anti-tumour response. Tumour-bearing mice were treated with different numbers of in vivo-activated, ovalbumin (OVA)-specific TCR^tg^ CD8^+^ T cells harvested from OT-I mice, either alone or in combination with lipid nanoparticle (LNP)-encapsulated mRNA encoding OVA. Tumour progression was monitored, and analyses were performed to assess survival, T cell expansion, phenotype, infiltration, and effector function. The combination therapy of a low dose of TCR^tg^ T cells and mRNA immunotherapy led to robust and durable anti-tumour responses, significantly improving survival compared to monotherapies. Notably, transferred TCR^tg^ T cells expanded only following mRNA immunotherapy, and circulating TCR^tg^ T cells exhibited a memory precursor and effector memory phenotype. These cells infiltrated tumours effectively and displayed more potent cytotoxic activity, resulting in regression of large tumours—even without prior lymphodepletion. Overall, our findings demonstrate that mRNA immunotherapy can substantially enhance the efficacy of TCR^tg^ T cell therapy in a solid tumour model. This combinatorial approach holds promise for overcoming key limitations of ACT by boosting T cell expansion, persistence, infiltration, and functional capacity within the tumour microenvironment.

**Supplementary Information:**

The online version contains supplementary material available at 10.1038/s41598-026-65120-4.

## Introduction

Adoptive T cell therapies (ACTs) are a set of immunotherapies that involve harvesting of T cells from a patient or a donor, ex vivo expansion (and occasionally engineering) and re-infusion into a patient, often together with interleukin 2 (IL-2) to support T cell expansion in vivo^1–3^. Current ACT techniques include tumour-infiltrating lymphocyte (TIL) therapy and transfer of genetically engineered T cells such as Transgenic T cell receptor (TCR^tg^) or Chimeric antigen receptor (CAR) T cell therapies^1^. TIL therapy uses a heterogeneous population of endogenous T cells extracted from a patient’s tumour, that are expanded ex vivo, and then re-infused into a lymphodepleted patient together with IL-2. This approach has shown promising results, particularly for the treatment of melanomas^1^. CAR T cell therapy employs genetically engineered T cells to express CARs that target specific antigens on the cancer cell surface, and has been shown to be effective in treating certain B cell malignancies^2,4^. TCR^tg^ T cell therapy utilises T cells expressing TCR^tg^ that recognise specific tumour epitopes presented on major histocompatibility complex (MHC) molecules^5^. In contrast to CAR T cells, TCR^tg^ T cells are also able to recognise epitopes derived from intracellular proteins, and can target a wide range of neoantigens and tumour-associated targets^3^.

In contrast to B cell malignancies, solid tumours are less accessible to adoptively transferred T cells. Solid tumours are often highly heterogenous and have an immunosuppressive microenvironment, hampering the efficacy of antigen-targeted therapies^3^. To date, only two ACTs have received FDA approval in solid malignancies; lifileucel, a TIL therapy for advanced melanoma^6^ and afamitresgene autoleucel, a melanoma-associated antigen-A4 (MAGE-A4)-targeted TCR^tg^ T cell therapy for advanced synovial sarcoma^7^.

In contrast, a rapidly expanding number of early‑phase clinical trials are evaluating TCR^tg^ T cell therapies for the treatment of solid malignancies, using diverse targets and engineering strategies. Early clinical activity has been demonstrated in studies targeting cancer‑testis antigens, most notably in the phase I trial NCT03686124, where PRAME‑directed TCR^tg^ T cell therapy (IMA203) achieved an overall response rate of ~ 52.5% (confirmed objective response rate [ORR] ~ 29%) across multiple solid tumours with manageable toxicity^8–10^. Building on these data, a randomized phase III study (NCT06743126) is currently evaluating IMA203 versus standard therapies in metastatic melanoma. In parallel, multiple ongoing phase I trials are exploring TCR^tg^ T cell approaches against shared oncogenic driver mutations, including TP53 R175H (NT‑175; NCT05877599) and KRAS G12D (e.g. NT‑112/AZD0240; NCT06218914), which aim to target intracellular neoantigens presented by defined HLA alleles in a broad range of solid tumours. Additional studies are expanding this modality to viral antigens such as HPV16 E7 (NCT05639972) in HPV‑associated cancers.

Nonetheless, several challenges remain associated with ACT. The first challenge is the difficulty of rapidly expanding sufficiently high numbers of T cells for infusion. Typically, the ex vivo expansion of the T cells can take up to one month, which may be too long for patients with fast-progressing disease^11^. To compensate for the transfer of a lower number of T cells, IL-2 injections are often administered after infusion to promote in vivo expansion of transferred T cells, but T cell expansion and persistence remain suboptimal for many patients^12^. Additionally, lymphodepletion is routinely performed before transfer to reduce the endogenous lymphocyte population and create a favourable environment for the infused T cells to expand and persist^13^. Unfortunately, patients frequently experience grade 3–4 adverse events when undergoing lymphodepletion and IL-2 administration, with toxicity observed in virtually all patients undergoing ACT^3,14^. Yet another challenge is the lack of durable cellular responses due to T cell exhaustion, anergy and clonal death, which often results in therapy failure after initial clinical response^15,16^.

The treatment of solid tumours with ACT faces additional challenges, including high antigenic heterogeneity and a complex immunosuppressive tumour microenvironment which can counteract the infiltration, functionality and fitness of T cells^1,2^.

Active and specific immunotherapies that stimulate transferred T cells by providing their cognate antigen are a potential adjunctive strategy to address the limitations and enhance the efficacy of ACT. To this end, successful examples include adenoviral, dendritic cell (DC) and mRNA-based immunotherapies^17^.

Combination of an antigen-encoding adenoviral immunotherapy, peri-tumour injections of non-coding immunostimulatory nucleic acids and TCR^tg^ T cell therapy led to complete regression of advanced melanoma in a lymphodepleted mouse model, suggesting that immunotherapy enhanced expansion and function of the transferred T cells^18^. DC immunotherapy targeting specific tumour antigens in combination with TCR^tg^ T or TIL has demonstrated efficacy in clinical trials of patients with melanoma and sarcoma^19–21^.

mRNA-based immunotherapy (ITh) has been or is currently studied in combination with CAR and TCR^tg^ T cell therapies. The combination of mRNA-based ITh and CAR-T cell therapy improved anti-tumour responses in mouse solid tumour models^22^. Based on this observation a clinical trial investigating Claudin-6 (CLDN6) CAR-T cells combined with a lipoplex-formulated mRNA encoding CLDN6 was performed in lymphodepleted patients (NCT04503278). Data from the phase 1/2 trial showed a 33% ORR and a 67% disease control rate across various CLDN6-positive solid tumours^23^.

For TCR^tg^ T cells, the combination of PRAME-directed TCR^tg^ T cells and an LNP-formulated mRNA encoding PRAME is currently being investigated in patients with melanoma and synovial sarcoma (NCT06946225,^24^). In this trial, patients receive lymphodepletion before and low doses of IL-2 after ACT.

Here, we investigate mRNA immunotherapy as a strategy to enhance the efficacy of TCR^tg^ T cell therapy in a murine colorectal cancer model expressing ovalbumin (OVA; MC38-OVA), with the rationale that an OVA-encoding mRNA immunotherapy could activate both transferred and endogenous OVA-specific T cells, thus enhancing anti-tumour efficacy in comparison with TCR^tg^ T cell monotherapy and potentially eliminating the need for lymphodepletion and IL-2 support.

## Materials and methods

### Animals

Female OT-I^tg^ C57BL/6 (CD45.2^+^) and congenic C57BL/6 CD45.1 mice of 6–12 weeks of age were acquired from Cyagen Biosciences and GemPharmatech. Animal experiments were conducted following protocols approved by the Institutional Animal Care and Use Committee (IACUC) of CrownBio. The care and use of animals was in accordance with the regulations of the Association for Assessment and Accreditation of Laboratory Animal Care (AAALAC). Animals were anaesthetised and euthanised using gradually increasing concentrations of CO_2_. Death was confirmed by cervical dislocation, in accordance with institutional animal care guidelines.

**Fig. 1:**
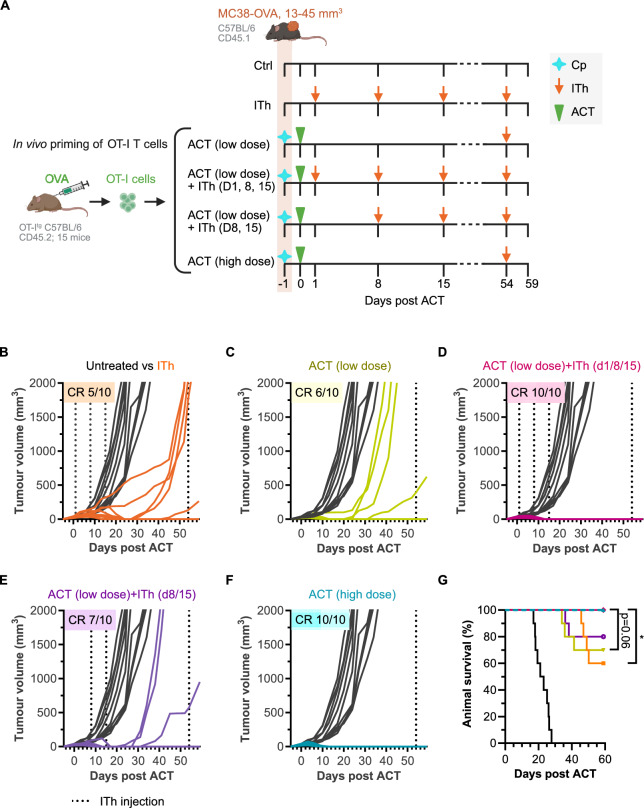
TCR^tg^ T cells in conjunction with mRNA immunotherapy generates a robust and sustained anti-tumour effect. (**A**) Experimental set-up. Therapies were initiated once small MC38-OVA tumours (13–45 mm^3^) had established. (**B**–**F**) Tumour volume (mm^3^) was assessed over time. Mice (n = 10/group) were treated with either (**B**) ITh on Days 1, 8 and 15, (**C**) a low dose of OT-I T cells (0.1 × 10^6^ cells/mouse), (**D**, **E**) a combination of low dose of OT-I T cells and ITh on Days 1, 8 and 15 or Days 8 and 15 or (**F**) a high dose of OT-I T cells (2 × 10^6^ cells/mouse). Surviving mice received an additional ITh treatment at Day 54. Tumour volume in untreated mice is shown in black. (**G**) Kaplan–Meier survival curves of the respective groups. Survival benefit was determined with the log-rank test. *p < 0.05. All mice that received OT-I T cells were lymphodepleted using Cp one day before cell transfer (Day -1). ACT: Adoptive transfer of pre-activated OT-I T cells, CR: complete response, D: Day, ITh: OVA-mRNA-LNP immunotherapy (i.m.), Cp: cyclophosphamide, A created with Biorender.com.

**Fig. 2:**
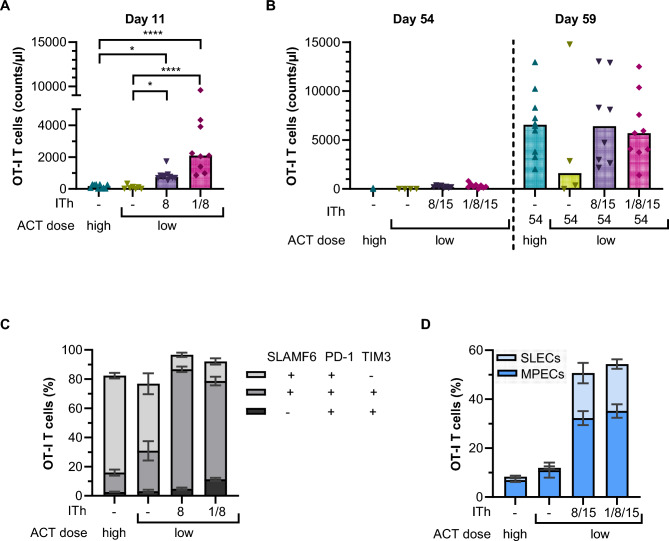
OT-I T cells expand following mRNA immunotherapy. (**A**, **B**) Effect of ITh administration on transferred OT-I T cell populations (CD45.2^+^ CD8^+^) in the blood at (**A**) Day 11 and (**B**) Days 54 and 59 post ACT. Number of TCR^tg^ cells are shown for individual mice (symbols) and the median (bars). (**C**) Frequencies of OT-I T cells expressing SLAMF6, PD-1 and TIM3 at Day 11 in the blood. (**D**) Frequencies of OT-I T cells with SLEC (KLRG1^+^ CD127^-^) and MPEC (KLRG1^-^ CD127^+^) phenotype at Day 40 in the blood. Data presented as mean ± SEM. ACT: Adoptive transfer of pre-activated OT-I T cells (high dose: 2 × 10^6^ cells/mouse, low dose: 0.1 × 10^6^ cells/mouse), d: Day, ITh: OVA-mRNA-LNP immunotherapy (i.m.), MPECs: memory precursor effector cells, SLECs: short-lived effector cells, SEM: standard error of the mean, Significance was assessed using the Kruskal–Wallis test followed by Dunn’s multiple-comparison test. * *p* < 0.05; **** *p* < 0.0001.

### Tumour challenge

**Fig. 3:**
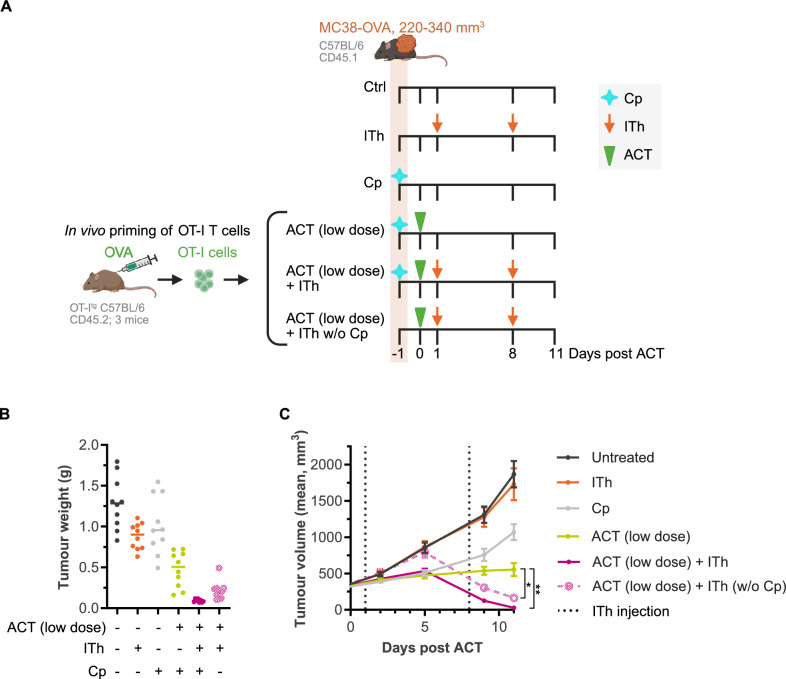
Combination of mRNA immunotherapy and TCR^tg^ T cells induces regression of large tumours, independent of prior lymphodepletion. (**A**) Mice (n = 10/group) bearing large MC38-OVA tumours (220–340 mm^3^; L = 0.91 cm x W = 0.7 cm to L = 1 cm x W = 0.7 cm) were treated with ITh and a low dose of OT-I T cells (0.1 × 10^6^ cells/mouse) as monotherapy or in combination. Some groups received lymphodepletion using cyclophosphamide one day prior to ACT as indicated. (**B**) Tumour weights for individual mice and median value/group on Day 11 post ACT and (**C**) tumour volume growth curves of treatment groups (mean ± SEM) are shown. ACT: Adoptive transfer of pre-activated OT-I T cells, Cp: cyclophosphamide, ITh: OVA-mRNA-LNP immunotherapy (i.m.), L: length, W: width. SEM: standard error of the mean, w/o: without. Significance was calculated with two-way ANOVA. * *p* < 0.05; ** *p* < 0.01, A created with Biorender.com.

After an acclimatisation period of 7 days, C57BL/6 CD45.1 mice were challenged subcutaneously with OVA-expressing murine colorectal cancer MC38-OVA cells (1 × 10^6^ cells/mouse) to the right rear flank. The MC38-OVA cell line was generated in the past by lentiviral transduction and expresses moderate amounts of OVA. Tumours were allowed to establish before initiation of treatment. Depending on the experimental set-up, treatment started when tumour volume was 13–45 mm^3^ (length [L] = 0.3 cm × width [W] = 0.3 cm to L = 0.64 cm × W = 0.37 cm), which was on Day 6 post tumour cell inoculation or 220–340 mm^3^ (L = 0.91 cm × W = 0.7 cm to L = 1 cm × W = 0.7 cm), which was on Day 16 post tumour cell inoculation, see Figs. 1A, 3A. Tumour-bearing mice were randomised before ACT according to tumour volume and body weights based on “Matched distribution” method (Study Director™ software, version 3.1.399.19, Studylog System, Inc., S. San Francisco, CA, USA). Tumour growth was measured twice per week with a calliper and tumour volume calculated as (length x width^2^)/2. Animals were monitored daily for morbidity and mortality.

### mRNA immunotherapeutic

The mRNA immunotherapeutic encoding full-length OVA comprised unmodified nucleotides and included the same non-coding elements as CV2CoV, a vaccine candidate against SARS-CoV-2^25^. The mRNA immunotherapeutic was encapsulated through a self-assembly process where an ethanolic lipid solution of an ionizable cationic lipid, 1,2-distearoyl-*sn*-glycero-3-phophocholine, cholesterol, and a polyethylene glycol-lipid was rapidly mixed with an aqueous solution of mRNA at acidic pH. The ionizable cationic lipid, proprietary to Acuitas Therapeutics (Vancouver, Canada) and LNP composition are described in patent applications WO 2017/075531A1 and WO 2017/004143, respectively. The average hydrodynamic diameter and polydispersity were measured by dynamic light scattering using a Zetasizer Nano ZS (Malvern Instruments Ltd., Malvern, UK) and encapsulation determined using a Quant-iT Ribogreen assay (Life Technologies). Mice were injected intramuscularly (i.m.) into the tibialis anterior at a dose of 1 µg of mRNA/mouse.

### Lymphodepletion and adoptive cell transfer (ACT)

Tumour-bearing mice were injected intraperitoneally (i.p.) with cyclophosphamide (Cp; 100 mg/kg, Selleck Chemicals) one day prior to ACT, unless otherwise stated. Cp was administered to induce lymphodepletion, mirroring clinical protocols used in ACT settings. For ACT, in vivo activated OT-I T cells were utilised. To achieve this, OT-I^tg^ mice were injected i.p. with OVA (0.5 µg, Sigma). Two days post injection, spleens were collected and CD8^+^ T cells isolated (STEMCELL) for subsequent ACT. The isolated OT-I T cells (0.1 × 10^6^ or 2 × 10^6^ cells/mouse) were delivered intravenously into Cp-treated or untreated tumour-bearing mice.

### FACS

Immune cells from spleen, blood and tumour samples were analysed by flow cytometry (fluorescence-activated cell sorting; FACS) using the reagents and antibodies listed in Supplementary Table S1 and S2. T cells specific for the OVA-derived peptide SIINFEKL were detected with T-Select H-2 Kb OVA Tetramer-SIINFEKL-APC (MBL). MPECs were defined as KLRG1^-^ CD127^+^ and SLECs as KLRG1^+^ CD127^-^.

For the detection of tumour-infiltrating lymphocytes, tumours were harvested at Day 11 post ACT and processed using a tumour dissociation kit (Miltenyi Biotec). Single cell suspensions were used for FACS staining, which started with Fc block (BD). Samples designated for tetramer staining were pre-treated with Dasatinib (Sigma) to prevent TCR internalization during tetramer incubation, followed by surface and intracellular antibody staining. For the latter, the FoxP3 Transcription Factor Staining Buffer Set (eBioscience) was used. Splenic T cells were isolated using GentleMACS (Miltenyi Biotec). Red blood cells were subsequently lysed using Red Blood Cell Lysis Buffer (BioGems). Splenic T cells were processed for FACS staining. For the detection of circulating T cells, peripheral blood was processed, followed by Fc block, Dasatinib treatment, antibody staining and red blood cell lysis. Further details can be found in the Supplementary Information.

### Statistical analysis

Survival time was analysed using the Kaplan–Meier method. The survival time was defined as the time from the day of randomisation until animal death or ethical endpoint. Kaplan–Meier curves were calculated for each group, and the log-rank test was used to compare survival curves between groups. For all other analyses, differences between groups were tested with two-way analysis of variance (ANOVA) or with Kruskal–Wallis test followed by Dunn’s multiple comparison test. Data are presented as individual values or as mean ± standard error of the mean (SEM) unless stated otherwise. GraphPad Prism version 10.0 was used for statistical tests and plots.

## Results

### Combination of TCR^tg^ T cells and mRNA immunotherapy induces an efficient and long-lasting anti-tumour response

The potential synergies between TCR^tg^ T cell therapy and an mRNA immunotherapeutic were studied in mice bearing small tumours expressing OVA (MC38-OVA, 13–45 mm^3^), that received either activated TCR^tg^ CD8^+^ T cells specific for the OVA peptide SIINFEKL (OT-I T cells), an mRNA immunotherapeutic encoding full-length OVA (ITh, OVA-mRNA-LNP), or both. To allow distinction between the transferred OT-I T cells, which express CD45.2, and endogenous T cells, congenic C57BL/6 CD45.1 mice were used as hosts.

OT-I T cells were transferred at two different doses: Transfer of a high dose of OT-I T cells (2 × 10^6^ cells) was performed only as monotherapy serving as positive control. Transfer of a low dose of OT-I T cells (0.1 × 10^6^ cells) was performed either as monotherapy or in combination with ITh to evaluate potential therapeutic benefit of the combination treatment. All mice that received ACT were lymphodepleted with cyclophosphamide (Cp) one day prior to ACT (Fig. 1A).

Monotherapy with either ITh or low-dose transfer of OT-I T cells to lymphodepleted mice resulted in complete response in 50% (5/10) and 60% (6/10) of mice, respectively (Fig. 1B, C). In contrast, combination of a low dose of OT-I T cells with ITh administration on Days 1, 8, and 15 led to complete eradication of tumours in all (10/10) mice (Fig. 1D). Combination of a low dose of OT-I T cells with ITh applications only on Days 8 and 15 demonstrated reduced efficacy, with complete eradication of tumours in 70% (7/10) of mice (Fig. 1E). The positive control group receiving a high dose of OT-I T cells achieved complete eradication of tumours in all (10/10) mice (Fig. 1F). Survival analysis showed 100% survival for mice receiving high-dose ACT or low-dose ACT and three ITh applications (on Days 1, 8 and 15), while late-escaping tumours were observed in other treatment groups during the 6-week observation period (Fig. 1G). These data demonstrate the synergistic potential of low-dose ACT and mRNA immunotherapy and highlight the requirement for early administration of mRNA immunotherapy post low-dose ACT. No adverse events were detected throughout the study in any treatment group, as evidenced by the absence of significant weight loss in mice (Supplementary Fig.S1).

### mRNA immunotherapy induces expansion of transferred TCR^tg^ T cells

Assessment of OT-I T cells in the blood on Day 11 of tumour-bearing mice demonstrated that a single administration of ITh on Day 8 after transfer of a low dose of OT-I T cells (0.1 × 10^6^ cells) resulted in a 4.6-fold higher number of OT-I T cells compared with mice receiving a low dose of OT-I T cells and a five-fold higher number of OT-I T cells compared to high-dose ACT (2 × 10^6^ cells) (Fig. 2A). The expansion of OT-I T cells was further increased after two administrations of ITh (on Days 1 and 8).

All ITh-treated mice received an additional ITh application on Day 15. Frequencies of the OT-I T cells declined in mice treated with ITh over time but were still detectable on Day 40 (Supplementary Fig. S2). OT-I T cells could be reactivated and expanded by a single administration of ITh on Day 54 in all groups (Fig. 2B). Interestingly, after administration of ITh on Day 54, expansion of OT-I T cells in mice that had received no prior mRNA immunotherapy, was observed despite cell numbers being almost non-detectable prior to ITh (Fig. 2B). These data indicate that administration of ITh post ACT has the potential to successfully expand low numbers of memory TCR^tg^ T cells consistent with long lasting memory responses. The magnitude of the expansion of the TCR^tg^ T cells (for example a factor 115 for high-dose ACT Day 54 to Day 59) strongly suggests that this expansion is primarily driven by the mRNA-encoded antigen and not an unspecific expansion of T cells due to mRNA-LNP induced cytokines.

Phenotypic characterisation of the OT-I T cells indicated that the combination therapy induced a phenotype characterised by co-expression of PD-1, TIM3 and SLAMF6 at Day 11 consistent with antigen‑experienced T cells showing activation-associated checkpoint expression at this early timepoint. Notably, SLAMF6 expression has been associated with progenitor-like, self-renewing T cells (Fig. 2C). In addition, combination therapy resulted in increased numbers of circulating memory precursor effector cells (MPECs) on Day 40 (Fig. 2D), which can contribute to long-lasting immunosurveillance.

### Combination of TCR^tg^ T cells and mRNA immunotherapy induces regression of large tumours in the absence of prior lymphodepletion

ACT without cyclophosphamide (Cp)-induced lymphodepletion did not have anti-tumour efficacy in the MC38-OVA model (Supplementary Fig. S3). As omitting lymphodepletion would be attractive due to its toxicity for patients^13^, an experiment was performed in MC38-OVA tumour-bearing mice to assess the efficacy of combined low-dose ACT and mRNA immunotherapy with or without prior lymphodepletion (Fig. 3A).

Therapy was initiated when tumours had reached volumes of 220–340 mm^3^, and despite the large initial tumour volume, combination therapy without prior Cp was sufficient to mediate prominent tumour shrinkage (Fig. 3B, C). The addition of Cp to combination therapy resulted in a modest improvement in efficacy, reflected by the smallest tumour volumes on Day 11, although the differences were not significant (Fig. 3C). In contrast, monotherapy with a low dose of OT-I T cells after Cp only stabilised tumour growth, while Cp alone resulted only in a delay of tumour growth compared with untreated and ITh monotherapy groups (Fig. 3C).

ITh monotherapy was not effective in reducing tumour volume, most likely due to the presence of large tumours and administration of only two ITh doses (Fig. 3B, C). However, when treatment was initiated in mice bearing small tumours, the mRNA immunotherapy could induce delay of tumour growth (Fig. 1B).

### Lymphodepletion supports the expansion of TCR^tg^ T cells but reduces endogenous T cell responses to ITh

**Fig. 4:**
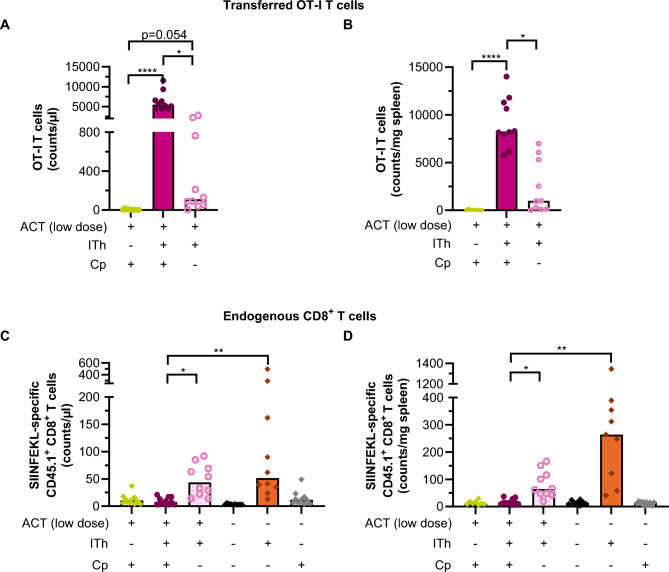
Lymphodepletion supports expansion of TCR^tg^ T cells after ACT and mRNA immunotherapy but suppresses endogenous CD8^+^ T cell responses. Mice with large MC38-OVA tumours were treated with ITh and a low dose of OT-I T cells (0.1 × 10^6^ cells/mouse) as monotherapy or in combination, with or without cyclophosphamide (n = 10/group). (**A**, **B**) Number of OT-I T cells (CD45.2^+^ CD8^+^) in the (**A**) blood and (**B**) spleen at Day 11 post ACT. (**C**, **D**) Number of endogenous SIINFEKL-specific CD8^+^ T cells (CD45.1^+^ CD8^+^ H-2Kᵇ OVA tetramer^+^) (**C**) in the blood and (**D**) in the spleen at Day 11 post ACT. Data presented for individual mice (symbols) and median values/group (bars). ACT: Adoptive transfer of pre-activated OT-I T cells, Cp: cyclophosphamide, ITh: OVA-mRNA-LNP immunotherapy (i.m.), Significance was assessed using the Kruskal–Wallis test followed by Dunn’s multiple-comparison test. * *p* < 0.05; ** *p* < 0.01; **** *p* < 0.0001.

Analysis of the blood and spleen revealed that lymphodepletion using Cp prior to ACT significantly increased the expansion of transferred OT-I T cells in mice receiving ACT and ITh (Fig. 4A, B). However, it also strongly reduced the induction of endogenous CD8^+^ T cell responses against the ITh mRNA-encoded antigen. While endogenous CD8^+^ T cells against SIINFEKL, the immunodominant CD8 epitope of OVA, were detected for MC38-OVA tumour bearing mice treated with ACT and ITh, these were absent in mice receiving additionally Cp (Fig. 4C, D). This effect might be caused by (i) the reduction in peripheral endogenous immune cells due to lymphodepletion (Supplementary. Fig. S4A, B) and (ii) potential interclonal competition between endogenous and transferred CD8⁺ T cells recognizing the same class I–restricted SIINFEKL epitope. Endogenous CD8^+^ T cells against SIINFEKL were also absent in MC38-OVA tumour bearing mice that received neither ITh nor Cp, demonstrating that expression of OVA by the tumours was insufficient to induce a CD8^+^ T cell response against this epitope.

This highlights the potential of ITh to not only expand transferred TCR^tg^ T cells but also to induce endogenous T cell responses against the encoded antigen in the ACT settings.

### mRNA immunotherapy increases the number of transferred TCR^tg^ T cells with cytotoxic potential in the tumours

**Fig. 5:**
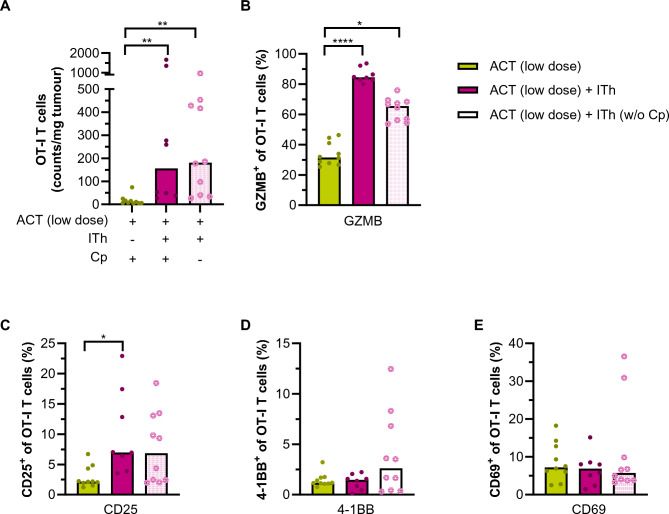
Combination of mRNA immunotherapy and low-dose ACT leads to increased intra-tumoural accumulation of TCR^tg^ T cells with enhanced cytotoxic potential. (**A**) Number of OT-I T cells (CD45.2^+^ CD8^+^) within the tumour upon low-dose ACT monotherapy (0.1 × 10^6^ cells/mouse) or combination treatment with ITh (injected Day 1 and 8), with or without lymphodepletion using cyclophosphamide. Expression of (**B**) Granzyme B, (**C**) CD25, (**D**) 4-1BB and (**E**) CD69 of intra-tumoural OT-I T cells was assessed by flow cytometry. Data presented for individual mice (symbols) and median value/group (bar). ACT: Adoptive transfer of pre-activated OT-I T cells, Cp: cyclophosphamide, GZMB: Granzyme B, ITh: OVA-mRNA-LNP immunotherapy (i.m.), Significance was assessed using the Kruskal–Wallis test followed by Dunn’s multiple-comparison test. * *p* < 0.05; ** *p* < 0.01; **** *p* < 0.0001.

To assess OT-I T cell infiltration, tumours were harvested on Day 11 and processed to generate single cell suspensions for analysis. The addition of mRNA immunotherapy significantly increased numbers of OT-I T cells within the tumours irrespective of prior Cp-induced lymphodepletion (Fig. 5A). Notably, the tumour-infiltrating CD8^+^ T cells demonstrated enhanced Granzyme B expression in mice treated with a combination of ACT and mRNA immunotherapy (regardless of prior lymphodepletion) compared with ACT alone, suggesting increased cytotoxic potential of the tumour-infiltrating OT-I T cells (Fig. 5B). Moreover, OT-I T cells showed an enhanced expression of the activation marker CD25 following combination therapy, whereas 4-1BB and CD69 expression of OT-I cells remained comparable in monotherapy and combination therapy groups (Fig. 5C–E).

## Discussion

Our results demonstrate that combining mRNA immunotherapy with TCR^tg^ T cell transfer more effectively suppressed tumour growth and improved survival in MC38‑OVA–bearing mice compared with either treatment alone. mRNA immunotherapy promoted expansion, reactivation, tumour infiltration and an effector phenotype of the transferred OT-I T cells. It also induced endogenous, antigen-specific CD8^+^ T cells, enhancing the OVA-specific immune response. Together, these data suggest that the addition of mRNA immunotherapy has the potential to overcome some of the main challenges of ACT therapy against solid tumours – even without prior lymphodepletion. Notably, full therapeutic efficacy required early initiation of mRNA immunotherapy, starting one day after low-dose ACT. While a delayed start at Day 8 led to comparable OT-I T cell expansion at later time points (Day 40 & Day 54), fast expansion of the transferred CD8^+^ T cells at early time points (e.g., Day 11) was likely critical for controlling the rapidly growing tumours.

The lack of durable efficacy represents one of the major challenges of ACT, reflected by frequent therapy failure after initial clinical responses^15^. Limited persistence of transferred T cells is one of the major factors contributing to relapse. In addition, conventional ex vivo expansion protocols, often relying on IL-2, may drive differentiation toward effector phenotypes and reduce the proportion of less differentiated, stem-like T cells, which have been associated with improved persistence and long-term responses^26^. Our findings suggest that these limitations may be mitigated by intermittent administration of LNP-encapsulated mRNA encoding the target antigen in the recipient in vivo, even at a late time point when memory T cells are present in very low numbers.

The tumour relapse observed following ITh or low-dose ACT monotherapy might be caused by limited persistence and/or progressive functional impairment of T cells, including exhaustion or anergy. In addition, recognition by antigen-specific CD8⁺ T cells could be impaired by the emergence of antigen-loss or MHC class I–deficient tumour variants.

Patients often receive IL-2 after ACT to expand the transferred T cells, which can be associated with severe systemic side effects caused by the activation of bystander host cells^12^. Prolonged IL-2 administration can also result in the expansion and activation of CD4^+^ Foxp3^+^ regulatory T cells, restricting administration to short term usage^27^. In our study, administration of mRNA immunotherapy led to a strong in vivo expansion of transferred T cells, even at later timepoints where minimal numbers of remaining TCR^tg^ T cells were observed, suggesting that the addition of mRNA immunotherapy has the potential to enhance survival and longevity of transferred T cells. Importantly, our results demonstrate that TCR^tg^ T cells derived from OT-I mice do not require IL-2 administration for mRNA immunotherapy-mediated expansion after adoptive transfer. If this finding extends to ex vivo engineered and expanded T cells currently used for ACT in the clinic, it could potentially reduce the risk of IL-2-associated toxicities.

The long-term clinical efficacy of ACT is frequently hampered by a loss of effector functions, as chronic stimulation during ex vivo expansion can lead to increased frequencies of dysfunctional or exhausted T cells^15^. mRNA immunotherapy can reactivate TCR^tg^ T cell effector function in vivo*.* Strong reactivation of expanded TCR^tg^ T cells was confirmed by the expression of PD-1 and TIM3, with additional SLAMF6 expression indicating that the cells had retained their self-renewal capacity. Interestingly, SLAMF6 expression in T cells has been found to correlate positively with checkpoint inhibitor response^28^, elevated immune activity and better prognosis in both breast cancer and melanoma^29^. In addition, an induction of a heterogenous pool of memory T cells, including persistent effector memory CD8^+^ T cells with the capability for fast expansion to antigen re-exposure and memory precursor cells was shown.

Effective T cell infiltration into solid tumours is crucial for ACT success^30^. In our study, we have presented evidence that mRNA immunotherapies can significantly increase the number of tumour-infiltrating TCR^tg^ T cells correlating with the enhanced number of TCR^tg^ T cells in the periphery. Importantly, the tumour-infiltrating TCR^tg^ T cells featured improved cytotoxic potential, as shown by increased Granzyme B expression, indicating that administration of mRNA immunotherapy may improve not only the quantity, but also the quality of the T cell response.

Tumours often escape immunotherapy by genetic or epigenetic suppression of antigen expression and presentation pathways^31^. As TCR^tg^ T cell therapy relies on presentation of a single target epitope, induction of intrinsic tumour escape mechanisms including emergence of antigen-loss variants or loss of epitope presentation by loss or down-regulation of the respective HLA class I allele expression represent formidable risks^31,32^. Addition of an mRNA immunotherapeutic encoding additional tumour antigens could hypothetically broaden the immune response beyond the antigen targeted by the TCR^tg^ T cells and potentially decrease the risk of immune evasion. Such additional tumour antigens might even be more efficient in priming endogenous T cells than the antigen recognized by the transgenic TCR because they avoid competition between naïve endogenous and transferred TCR^tg^ T cells, an effect which likely explains the lower induction of endogenous SIINFEKL-specific CD8^+^ T cells by ITh in the presence of ACT (Fig. 4C, D). In the current work we observed induction of endogenous CD8^+^ T cell responses against the immunodominant CD8 epitope of the mRNA-encoded antigen OVA in mice without lymphodepletion, while potentially increased breadth of the response remains to be shown in future studies.

Before receiving ACT, patients undergo lymphodepleting chemotherapy, potentially causing long-term cytopenia^33^. Prolonged systemic side effects such as grade 3 or 4 anaemia, thrombocytopenia, neutropenia, leukopenia, or lymphopenia, and an increased infection risk are common consequences of lymphodepleting chemotherapy^3^. The main aim of lymphodepletion is the reduction of endogenous lymphocytes to improve engraftment, homing and survival of transferred T cells^13^. Indeed, we observed prominent expansion of transferred T cells only when ACT was combined with mRNA immunotherapy in lymphodepleted hosts. We also observed that lymphodepletion was strictly required for tumour growth inhibition when mice were treated with ACT monotherapy. These observations are consistent with recent work^34^ demonstrating that lymphodepleting preconditioning enhances expansion of transferred T cells and promotes elimination of primary tumours, but at the same time compromises host antitumor immunity and reduces protection against antigen-loss variants that are resistant to ACT. Accordingly, while mRNA immunotherapy induced endogenous T cell responses against the encoded antigen in our study, these responses were markedly reduced following lymphodepletion prior to ACT. Interestingly, our data demonstrates that robust anti-tumour responses against large tumours can be achieved by combining TCR^tg^ T cell therapy and mRNA immunotherapy without lymphodepletion. As such, our data suggests that addition of mRNA immunotherapy to ACT may eliminate the need for lymphodepletion, thereby reducing severe ACT-induced side effects, as well as the observed reduction of mRNA-induced endogenous T cell responses in mice receiving combination therapy with lymphodepletion.

Finally, the timely generation of sufficient numbers of TCR^tg^ T cells to achieve effective treatment remains a major challenge^14^. In this study, combining a low dose of TCR^tg^ T cells with mRNA immunotherapy resulted in sustained therapeutic efficacy, suggesting that mRNA immunotherapy may help overcome this limitation by enabling the infusion of lower T‑cell numbers followed by in vivo expansion of the transferred cells.

Our preclinical observations in a murine model highlight the potential of combining TCR^tg^ T cell therapy with mRNA-based ITh. Further studies are required to validate these findings in the human setting and to assess their applicability to clinically established TCR^tg^ T cell therapy protocols. To this end the current study has several limitations. First, the model employed TCR^tg^ CD8⁺ T cells derived from transgenic mice following in vivo activation, in contrast to the ex vivo engineered, expanded and polyclonal human T cells used in clinical practice. Second, the engineered MC38‑OVA model may not fully capture the heterogeneity and antigen amounts observed in clinical settings. Nonetheless, as the tumour cells express only moderate amounts of OVA that alone do not induce measurable SIINFEKL-specific CD8^+^ T cell responses, they can serve as a valuable model. Third, the SIINFEKL peptide targeted in this system exhibits high affinity for both the presenting MHC molecule and the transgenic OT‑I TCR; therefore, additional target antigens and tumour models should be evaluated to confirm generalizability. Fourth, while the capacity of ITh to induce T cell responses specific to the encoded tumour antigens has been well established^35^, further studies using mRNAs encoding antigens not recognized by the transferred TCR^tg^ would help to substantiate this effect in the context of TCR^tg^ T cell therapy. Finally, the pronounced expansion of antigen-specific OT-I T cells following mRNA immunotherapy supports a dominant role for antigen-driven stimulation. However, as an irrelevant mRNA control was not included in the experimental design it cannot be excluded that innate immune activation induced by the mRNA–LNP formulation contributes to the observed therapeutic effects. Future studies incorporating control mRNAs will be required to delineate the relative contributions of antigen-specific versus antigen-independent mechanisms.

## Conclusion

The presented data demonstrate that mRNA immunotherapy enhances the efficacy of TCR^tg^ T cell therapy and promotes durable antitumour responses. Our findings indicate multifactorial benefits of combining mRNA immunotherapy with TCR^tg^ T cells, including enhanced in vivo expansion and reactivation of transferred T cells, sustained effector function, induction of memory T cell populations, and increased tumour infiltration and cytotoxic activity. Notably, mRNA immunotherapy also mediated antitumour efficacy in the absence of lymphodepletion.

## Supplementary Information

Supplementary Information.

## Data Availability

All data supporting the findings of this study are available within the paper and its Supplementary Information.

## Abbreviations

ACT: Adoptive T cell therapy
ANOVA: Analysis of variance
CAR: Chimeric antigen receptor
Cp: Cyclophosphamide
CR: Complete response
D: Day
DC: Dendritic cell
FACS: Fluorescence-activated cell sorting
FDA: The United States Food and Drug Administration
GZMB: Granzyme B
IL-2: Interleukin 2
i.m.: Intramuscular
i.p.: Intraperitoneal
ITh: OVA mRNA LNP immunotherapy
LNP: Lipid nanoparticle
MHC: Major histocompatibility complex
mRNA: Messenger ribonucleic acid
n: Number
OVA: Ovalbumin
SEM: Standard error of the mean
TCR: T cell receptor
tg: Transgenic
TIL: Tumour-infiltrating lymphocytes
