# Supplementary material for: mRNA-based immunotherapy improves the efficacy of adoptively transferred TCR transgenic T cells in a murine solid tumour model
Source: Sci Rep. 2026 Aug 8;16:24539. doi: 10.1038/s41598-026-65120-4 (PMC13452821; doi:10.1038/s41598-026-65120-4)
Supplement: Supplementary file 1 [file 41598_2026_65120_MOESM1_ESM.docx]

**Supplementary materials**

**Supplementary Figure S1. Combination of mRNA immunotherapy and TCR^tg^ T-cell therapy did not result in severe weight loss of MC38‑OVA-bearing mice.** Time-course of body weight change of MC38‑OVA bearing mice in ITh and OT‑I ACT monotherapy (high dose: 2 x 10^6^ cells/mouse, low dose: 0.1 x 10^6^ cells/mouse) and combination therapy groups (n=10/group). Mice that received ACT underwent lymphodepletion using cyclophosphamide one day prior to ACT. Data presented as mean ± SEM.

ACT: Adoptive transfer of pre-activated OT‑I T cells D: Day, ITh: OVA‑mRNA‑LNP immunotherapy (i.m.), SEM: standard error of the mean.

**Supplementary Figure S2. Addition of mRNA immunotherapeutic to TCR^tg^ T-cell therapy results in sustained expansion of transferred OT-I T cells.** Number of OT‑I T cells (CD45.2^+^ CD8^+^) in the blood at Day 40 was measured in mice that received a high dose of OT-I T cells (n=10, 2 x 10^6^ cells/mouse), a low dose of OT-I cells (n=7, 0.1 x 10^6^ cells/mouse) and those treated with a combination of low dose ACT and ITh, either administered on Days 8 and 15 (n=10) or on Days 1, 8 and 15 (n=9). Mice that received ACT were treated with cyclophosphamide one day prior to ACT.

ACT: Adoptive transfer of pre-activated OT‑I T cells, ITh: OVA‑mRNA‑LNP immunotherapy (i.m.), Significance was assessed using the Kruskal–Wallis test followed by Dunn’s multiple‑comparison test. ** p<0.01; *** p<0.001

**Supplementary Figure S3. TCR^tg^ T-cell therapy monotherapy without lymphodepletion does not have anti-tumour efficacy.** MC38‑OVA tumour volume growth curves of mice (n=8/group) treated with different doses of transferred OT‑I T cells (1 x 10^6^; 2 x 10^5^ or 5 x 10^4^ cells/mouse) without prior lymphodepletion using cyclophosphamide. Data presented as mean ± SEM per group per time point.

ACT: Adoptive transfer of pre-activated OT‑I T cells, Cp: cyclophosphamide, SEM: standard error of the mean.

**Supplementary Figure S4. Lymphodepletion with cyclophosphamide reduces the number of endogenous T cells.** Number of (A) endogenous immune cells (CD45^+^) and (B) endogenous T cells (CD45.1^+^ Thy1.2^+^) in the blood at Day 1 in mice two days post cyclophosphamide treatment (n=40) or mice without cyclophosphamide treatment (n=20). Bar graphs show Median.

Cp: cyclophosphamide, n: number of mice

**Supplementary Fig.S5**

**Supplementary Figure S5. Gating strategy for discriminating endogenous CD8^+^ T cells vs transferred OT-I T cells and SIINFEKL-specific endogenous CD8^+^ T cells.** The gating strategy used to distinguish endogenous (CD45.1^+^) CD8^+^ T cells from transferred (CD45.2^+^ CD8^+^) OT-I T cells, as well as to identify SIINFEKL‑specific endogenous CD8⁺ T cells using the H‑2Kᵇ OVA tetramer, is shown. Counting beads were added according to the manufacturer’s instructions and were identified based on their SSC‑A/FSC‑A profile.

**Supplementary Fig.S6**

FMO CD45.1

FMO CD45.2

FMO H‑2Kᵇ OVA tetramer

**Supplementary Figure S6. Fluorescent-minus-one (FMO) controls for gating endogenous vs transferred CD8^+^ T cells and SIINFEKL-specific endogenous CD8^+^ T cells.** FMO controls have been applied to gating strategy for identifying endogenous vs transferred CD8^+^ T cells and SIINFEKL-specific endogenous CD8^+^ T cells.

**Supplementary Table S1. Reagents**

4

| **Reagents** | **Vendor** | **Catalogue** |
| --- | --- | --- |
| Mouse BD Fc Block™ | BD Biosciences | 553141 |
| IC fixation buffer | eBioscience | 00-8222-49 |
| 10X RBC Lysis Buffer | Bio-gems | 64010-00-100 |
| ACK lysing buffer | Invitrogen | A1049201 |
| Brefeldin A, BFA | MULTI Sciences | CS0003 |
| 123count eBeads | eBioscience | 01-1234-42 |
| Murine tumor dissociation kit | Miltenyi Biotec | 130-096-730 |
| Dasatinib | Sigma | SML2589-50MG |
| Foxp3/Transcription Factor Staining Buffer Set | eBioscience | 00-5523-00 |
| L/D (APC-eFluor 780) | eBioscience | 65-0865-18 |

ACK: Ammonium-Chloride-Potassium, IC: intracellular, L/D: Live/Dead, RBC: red blood cell

**Supplementary Table S2. Antibodies for flow cytometry**

| **Antibodies** | **Fluorochrome** | **Clone** | **Cat.** | **Vendor** |
| --- | --- | --- | --- | --- |
| Slamf6/Ly108 | APC | 330-AJ | 134610 | BioLegend |
| CD45.2 | BV421 | 104 | 109832 | BioLegend |
| CD45.2 | BV785 | 104 | 109839 | BioLegend |
| CD8 | FITC | 53-6.7 | 100706 | BioLegend |
| CD4 | APC | GK1.5 | 100412 | BioLegend |
| PD-1 | BV605 | 29F.1A12 | 135220 | BioLegend |
| Tim3 | PE | RMT3-23 | 119703 | BioLegend |
| CD45.1 | APC | A20 | 110714 | BioLegend |
| CD45.1 | BUV395 | A20 | 565212 | BD |
| CD44 | BV510 | IM7 | 103043 | BioLegend |
| CD62L | BV785 | MEL-14 | 104440 | BioLegend |
| CD127 | PE | S18006K | 158204 | BioLegend |
| KLRG1 | APC | 2F1 | 138412 | BioLegend |
| Thy1.2 | PerCP-Cy5.5 | 30-H12 | 105338 | BioLegend |
| H-2Kb OVA Tetramer-SIINFEKL | APC | - | TB-5001-2C | MBL |
| Granzyme B | PE/Cy7 | QA16A02 | 372214 | BioLegend |
| CD69 | AF700 | H1.2F3 | 104539 | BioLegend |
| CD25 | BV605 | PC61 | 102036 | BioLegend |
| 4-1BB | BV650 | 17B5 | 756685 | BD |

**Supplementary Methods**

**Spleen processing and stimulation**

Spleens were harvested and dissociated using GentleMACS (Miltenyi Biotec) according to manufacturer’s instructions. The cell suspension was centrifuged at 4°C and 1500 rpm for 10 min. The pellet was resuspended in 5 ml ACK red blood cell lysis buffer for 5 min at room temperature according to the manufacturer’s instructions. Lysis was stopped by adding ice‑cold RPMI 1640 medium to a final volume of 40 ml, followed by centrifugation. The cell pellet was then resuspended in 10 ml ice‑cold RPMI 1640 medium and passed once more through a 70 µm strainer prior to cell counting. Cells were processed for surface and intracellular staining and subsequently analysed by flow cytometry.

**Blood processing and general staining procedure**

Whole blood was collected at defined time points and transferred into a 96‑well plate. Samples were centrifuged at 4°C for 10 min at 1500 rpm. The resulting cell pellet was resuspended in staining buffer containing 1 µg/ml Fc‑block and incubated for 10 min at 4°C. Subsequently, the surface antibody master mix—prepared in Fc‑blocking buffer—was added, and samples were incubated for 30 min at 4 °C.

For blood samples and unstimulated splenocytes, 2 ml of pre‑warmed 1× red blood cell lysis buffer was added, followed by incubation for 10 min at room temperature in the dark. Afterward, 2 ml wash buffer were added, and samples were centrifuged at 300 × g for 5 min. This wash step was repeated once.

Cell pellets were then resuspended in Fixation/Permeabilization working solution and incubated for 30 min at room temperature according to the manufacturer’s instructions, followed by centrifugation. Cells were washed twice with permeabilization buffer before intracellular staining for 30 min. After staining, cells were washed twice with permeabilization buffer and once with FACS buffer. Finally, cells were resuspended in FACS buffer and acquired. An example gating strategy can be seen in Supplementary Fig.S5 and 6.

For blood samples, counting beads were added immediately prior to acquisition on the flow cytometer (Supplementary Fig.S5).

**Tumour dissociation**

Tumour tissues were dissociated using the Tumour Dissociation Kit (Miltenyi Biotec) according to the manufacturer’s instructions. Briefly, tumours were collected, weighed, and cut into small fragments of approximately 2–4 mm. The pieces were transferred into gentleMACS C‑tubes containing the appropriate enzyme mix. After completion of the gentleMACS dissociation program, the samples were resuspended and passed through a 70 µm cell strainer. The resulting single‑cell suspensions were subsequently used for cell counting and downstream applications.

**Dasatinib pretreatment**

Samples designated for tetramer staining were pre-treated with Dasatinib (Sigma) to prevent TCR internalization and thereby enhance tetramer staining. In detail, 5 µl of a 0.55µM Dasatinib solution were added to 100 µl of the respective samples and incubated at 37°C for 30 min prior to tetramer incubation.
